# Effectiveness of a Mindfulness Meditation App Based on an Electroencephalography-Based Brain-Computer Interface in Radiofrequency Catheter Ablation for Patients With Atrial Fibrillation: Pilot Randomized Controlled Trial

**DOI:** 10.2196/44855

**Published:** 2023-05-03

**Authors:** Ying He, Zhijie Tang, Guozhen Sun, Cheng Cai, Yao Wang, Gang Yang, ZhiPeng Bao

**Affiliations:** 1 Department of Cardiology The First Affiliated Hospital of Nanjing Medical University Nanjing China; 2 School of Nursing Nanjing Medical University Nanjing China

**Keywords:** atrial fibrillation, radiofrequency catheter ablation, mindfulness meditation, brain computer interface, mHealth, smartphone app, randomized controlled trial

## Abstract

**Background:**

Radiofrequency catheter ablation (RFCA) for patients with atrial fibrillation (AF) can generate considerable physical and psychological discomfort under conscious sedation. App-based mindfulness meditation combined with an electroencephalography (EEG)-based brain-computer interface (BCI) shows promise as effective and accessible adjuncts in medical practice.

**Objective:**

This study aimed to investigate the effectiveness of a BCI-based mindfulness meditation app in improving the experience of patients with AF during RFCA.

**Methods:**

This single-center pilot randomized controlled trial involved 84 eligible patients with AF scheduled for RFCA, who were randomized 1:1 to the intervention and control groups. Both groups received a standardized RFCA procedure and a conscious sedative regimen. Patients in the control group were administered conventional care, while those in the intervention group received BCI-based app–delivered mindfulness meditation from a research nurse. The primary outcomes were the changes in the numeric rating scale, State Anxiety Inventory, and Brief Fatigue Inventory scores. Secondary outcomes were the differences in hemodynamic parameters (heart rate, blood pressure, and peripheral oxygen saturation), adverse events, patient-reported pain, and the doses of sedative drugs used in ablation.

**Results:**

BCI-based app–delivered mindfulness meditation, compared to conventional care, resulted in a significantly lower mean numeric rating scale (mean 4.6, SD 1.7 [app-based mindfulness meditation] vs mean 5.7, SD 2.1 [conventional care]; *P*=.008), State Anxiety Inventory (mean 36.7, SD 5.5 vs mean 42.3, SD 7.2; *P*<.001), and Brief Fatigue Inventory (mean 3.4, SD 2.3 vs mean 4.7, SD 2.2; *P*=.01) scores. No significant differences were observed in hemodynamic parameters or the amounts of parecoxib and dexmedetomidine used in RFCA between the 2 groups. The intervention group exhibited a significant decrease in fentanyl use compared to the control group, with a mean dose of 3.96 (SD 1.37) mcg/kg versus 4.85 (SD 1.25) mcg/kg in the control group (*P*=.003).The incidence of adverse events was lower in the intervention group (5/40) than in the control group (10/40), though this difference was not significant (*P*=.15).

**Conclusions:**

BCI-based app–delivered mindfulness meditation effectively relieved physical and psychological discomfort and may reduce the doses of sedative medication used in RFCA for patients with AF.

**Trial Registration:**

ClinicalTrials.gov NCT05306015; https://clinicaltrials.gov/ct2/show/NCT05306015

## Introduction

Atrial fibrillation (AF) is recognized as the most common cardiac arrhythmia worldwide, with an estimated prevalence of 2%-4% in adults [1]. Epidemiological studies indicate that AF increases the risk of stroke by 5-fold and the risk of overall mortality by 3.5-fold [2,3]. AF is becoming an increasingly extensive public health problem and causes substantial health economic burden [4-6]. Radiofrequency catheter ablation (RFCA) has become a first-line therapy for AF to improve symptoms, cardiac function, and quality of life, and has been shown to be cost-effective [7-9]. In China, the number of patients with AF is estimated to be approximately 20 million, and the number of RFCAs for AF exceeds 30,000 every year [10,11].

Considering the longer general anesthesia preparation time, higher economic costs, and potential complications, conscious sedation is used for RFCA at most centers in China [12-14]. However, even under well-tolerated doses of sedative drugs, sedation-related side effects such as nausea, vomiting, and oversedation are common [15]. Furthermore, patients are required to remain motionless and endure radiofrequency energy burning their myocardia for hours during the complex RFCA procedure. Consequently, even under deep sedation, patients may still experience considerable pain, anxiety, fatigue, and other discomforts, which may be associated with poor outcomes [16-18]. Several studies have indicated that nonpharmacological interventions could be ideal adjuncts to sedative drugs, effectively reducing patients' physical or psychological discomfort and the required doses of sedative drugs during medical invasive procedures [19,20].

Mindfulness meditation originates from Buddhist teachings and refers to a category of techniques used to pay attention to the present moment and accept all that arises without judgment [21]. Numerous studies suggest that mindfulness meditation may hold potential for alleviating pain, fatigue, and negative emotions [22,23]. However, the effects of mindfulness meditation during AF ablation remain uncertain. In recent years, with the rapid development of digital medicine, app-based mindfulness interventions have been preliminarily shown to be effective and accessible [24].

A brain-computer interface (BCI) is defined as a technology for establishing external information communication and control pathways between the human brain and computers or other electronic devices [25]. Electroencephalography (EEG) is a conventional form of brain signal acquisition, which can be recognized and reflected (usually through visual or auditory signals) by BCI [26]. Studies have shown that EEG-based BCI devices can sense and classify human psychological states, which may facilitate mindfulness meditation practice [27,28].

This study aimed to determine the effects of a BCI-based mindfulness meditation app on RFCA for patients with AF. The primary hypothesis was that the intervention group would experience significant improvements in perceived pain, anxiety, and fatigue compared to the control group. We also hypothesized that the intervention might decrease the use of sedative drugs and the incidence of adverse events.

## Methods

### Study Design

This was a single-center, 2-arm, parallel-group, prospective, pilot randomized controlled trial. Patients were randomized 1:1 to the intervention and control groups using a computer-generated randomization list. Due to the nature of the study design, neither program implementers nor patients could be blinded to the intervention. However, the investigators performing the outcome assessments and data analysis were blinded to the group allocation.

### Participants

#### Overview

Patients were eligible for the study if they were (1) diagnosed with AF, (2) at least 18 years old, (3) undergoing their initial RFCA procedure, and (4) willing to participate in the study. Patients were excluded if they had (1) severe systemic diseases such as malignant tumors, (2) a history of mental illness and cognitive complaints, and (3) difficulty understanding the questionnaire and the study aims. They were also excluded if they experienced drastic changes in their condition during RFCA.

Sample size calculations were conducted using PASS 2021 (NCSS LLC) software and based on previous studies [29,30]. Power analysis showed that a sample size of 70 participants was sufficient to have 90% statistical power at a 2-sided α of .05 for significance. To account for a 20% loss rate, the planned sample size was 84 participants.

All patients underwent a standardized RFCA procedure for AF with a 3D mapping system (CARTO 3, Biosense Webster) and were provided with standardized information about the study and the potential benefits and risks of the interventions. Both groups received the same sedative regimen during RFCA, which was adjusted by the interventional physician based on the patient's response to the medication and reported pain levels. The regimen included a single dose of parecoxib (40 mg), fentanyl (1 mcg/kg/hour), and dexmedetomidine as necessary, with dosing adjustments made in accordance with standard pain management procedures at our institution. The fentanyl maintenance infusion rate ranged from 0 to 2 mcg/kg/hour, while the dexmedetomidine maintenance infusion rate ranged from 0 to 1 mcg/kg/hour.

#### Intervention Group

**Figure 1:**
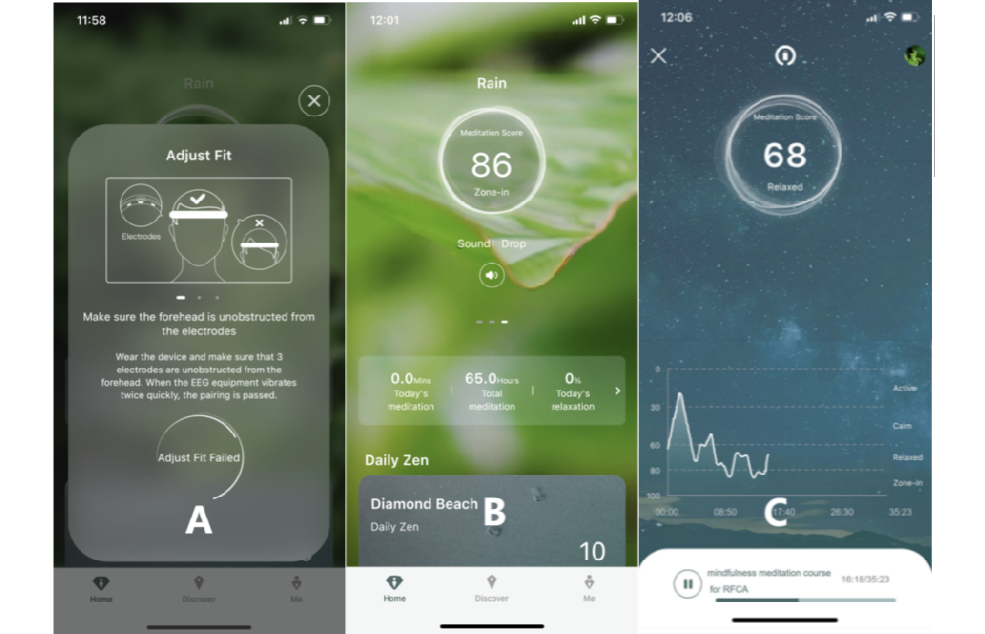
Screenshots of the study app. (A) Device connection, (B) background sound setting, and (C) mindfulness meditation course).

Patients in the intervention group received mindfulness meditation guidance delivered through a Chinese-language interface and voice app (Focus Zen, version 2.1.1) along with a BCI-based headband. A mobile phone and a Samsung tablet device with the preinstalled app were prepared in the cardiac catheterization laboratory. Before ablation, study staff briefly introduced the method and meaning of mindfulness meditation to help patients understand the intervention content. Mindfulness meditation represents a practice of awareness in which the person gradually and purposefully focuses on the present without judgement to achieve a state of deep relaxation [31]. The app's developers designed a 35-minute mindfulness meditation course specifically for patients with AF to help them relax during ablation without affecting the ablation procedure (eg, the course instructed patients to breathe evenly rather than deeply). We provided patients with Bluetooth earphones and set the background sound within the app in accordance with the patients' preferences, such as forest, beach, or rain sounds. During the mindfulness meditation practice, patients were guided by a female voice through the app to relax muscles, regulate breathing, and practice visualization and body scanning. Simultaneously, the app collected EEG information using a headband device that was synchronized with the app through Bluetooth technology (Figure 1). An artificial intelligence algorithm included in the app was used to analyze the EEG data and classify the patient's brain state as active, calm, relaxed, or meditative. The app interface and headband light color were adjusted in accordance with the patients' state. Additionally, the app prompted the patients' current brain state through background sound effects and guided the patient to maintain the state or make adjustments through the app voice.

#### Control Group

The control group received routine care for their ablation procedure and was informed about the procedure of ablation and characteristics of impending pain in ablation, as in the intervention group. Psychological and supportive care were provided in accordance with the patients' needs. However, patients in the control group wore the headband device without using the earphones and did not receive mindfulness meditation guidance provided by the app.

### Outcome Measurements

Both the intervention and control groups were administered 2 surveys, one 30 minutes before ablation and one within 30 minutes after ablation, to assess pain intensity, fatigue, and anxiety using specific paper questionnaires. Demographic information and characteristics of participants were collected at baseline. The study staff recorded patients' hemodynamic parameters (heart rate, blood pressure, and peripheral oxygen saturation [SpO_2_]), spontaneously reported pain, the doses of sedative drugs used, and adverse events during ablation.

The primary outcomes were pain and anxiety levels during ablation and fatigue severity after ablation. The intensity of pain was measured using the numeric rating scale, with scores that ranged from 0 (no pain) to 10 (the worst possible pain) [32,33]. The State Anxiety Inventory (A-State) is a subscale of the State-Trait Anxiety Inventory [34], which is mainly used to assess the anxiety state in a specific situation. The A-State score was used in this study to explore the anxiety level of patients during ablation. We evaluated the patients' fatigue after ablation using the Brief Fatigue Inventory (BFI) [35], a 10-item validated scale. A higher score indicates a greater level of fatigue.

Secondary outcomes included mean heart rate, blood pressure, and SpO_2_ during ablation. Adverse events were defined as excessive fluctuations in blood pressure (fluctuations of >50 mm Hg in systolic blood pressure), nausea and vomiting, and vasovagal reaction. The study staff also recorded the number of times of spontaneously reported pain and the doses of sedative drugs used during ablation.

### Statistical Analysis

Statistical analysis was performed using SPSS (version 22.0; IBM Corp) and based on the intention-to-treat principle with a 2-sided significance level of .05. Data were analyzed using descriptive statistics and checked for the normality of their distribution. Descriptive continuous variables are presented as mean (SD) values and categorical variables as frequency and percentage values. Differences between study groups were analyzed using an independent 2-sample *t* test for numerical variables and the Mann-Whitney *U* test, chi-square test, or the Fisher exact test for categorical variables.

### Ethical Considerations

This study was conducted at the cardiac catheterization laboratory of the First Affiliated Hospital of Nanjing Medical University, Nanjing, China, from April to September 2022. All study patients provided oral or written informed consent. This study was approved by the ethics committee of the First Affiliated Hospital of Nanjing Medical University (2022-SR-086) and registered at ClinicalTrials.gov (NCT05306015). Procedures were conducted in accordance with the tenets of the Declaration of Helsinki.

## Results

### Baseline Characteristics

**Figure 2:**
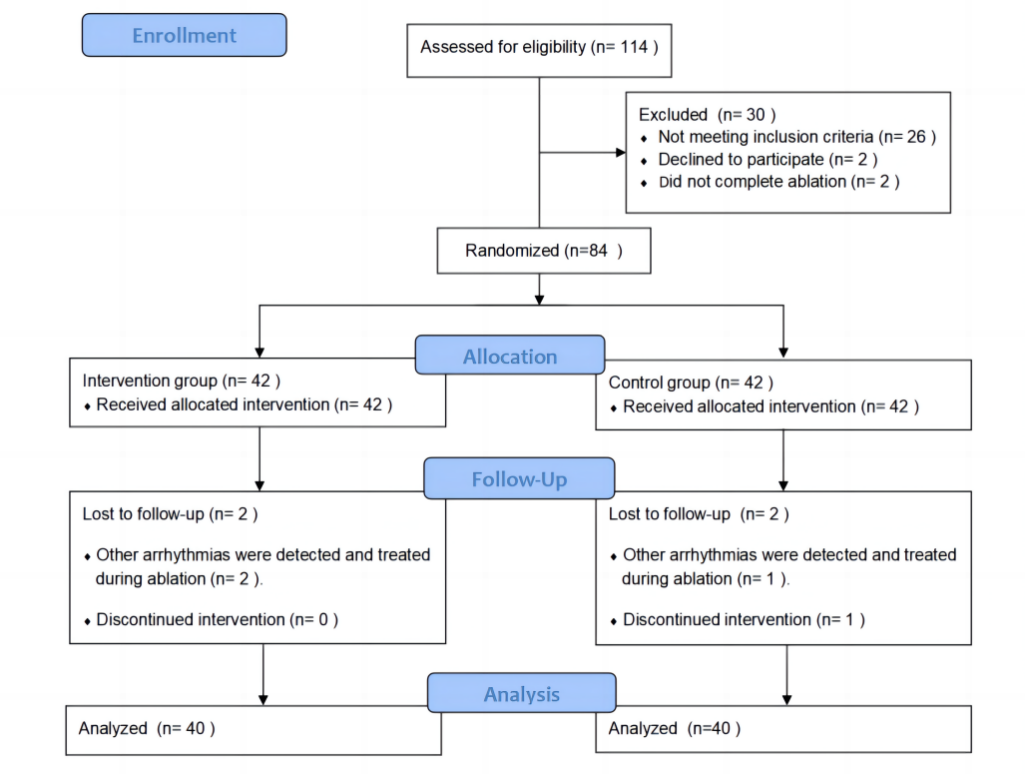
CONSORT (Consolidated Standards of Reporting Trials) flow diagram.

A total of 84 patients (42 patients each in the intervention and control groups) were enrolled and completed baseline measures in this study. A total of 4 patients (2 each from the intervention and control groups) were excluded from the study. Figure 2 shows the CONSORT (Consolidated Standards of Reporting Trials) diagram for this clinical trial.

No significant differences were found between the intervention group and the control group in baseline characteristics (Table 1). Neither group had previous experience with practicing mindfulness meditation.

**Table 1:** Baseline characteristics of the study participants (N=80).

Characteristics		Intervention group (n=40)	Control group (n=40)	*P* value	
Age (years), mean (SD)		58.1 (10.4)	60.0 (11.2)	.44	
Gender (female), n (%)		15 (38)	16 (40)	.82	
Weight (kg), mean (SD)		71.3(13.6)	69.5(11.2)	.53	
BMI (kg/m²), mean (SD)		24.9 (3.0)	24.9 (3.3)	.97	
LAD^a^ (mm), mean (SD)		40.6 (6.5)	40.8 (4.2)	.89	
LVEF^b^ (%), mean (SD)		62.0 (5.9)	60.9 (6.4)	.40	
Hypertension, n (%)		14 (35)	16 (40)	.64	
DM^c^, n (%)		6 (15)	4 (10)	.50	
CHD^d^, n (%)		3 (8)	9 (23)	.06	
**Type of AF^e^, n (%)**					.37
	Paroxysmal	24 (60)	20 (50)		
	Persistent	16 (40)	20 (50)		
**NYHA^f^ class, n (%)**					.59
	Class I	32 (80)	30 (75)		
	Class II	8 (20)	10 (25)		
RFCA^g^ time (minutes), mean (SD)		40.1 (14.3)	42.6 (15.0)	.44	
RFCA energy (Watts), mean (SD)		42.2 (3.8)	42.1 (3.6)	.81	
RFCA temperature (℃), mean (SD)		28.8 (3.3)	29.5 (4.3)	.39	

^a^LAD: left atrium diameter.

^b^LVEF: left ventricular ejection fraction.

^c^DM: diabetes mellitus.

^d^CHD: coronary heart disease.

^e^AF: atrial fibrillation.

^f^NYHA: New York Heart Association.

^g^RFCA: radiofrequency catheter ablation.

### Primary Outcomes

We found no significant difference in the baseline pain, anxiety, and fatigue scores between the intervention and control groups (Table 2). After the intervention, compared to the control group, there were significant differences in numeric rating scale (mean 4.6, SD 1.7 [intervention group] vs mean 5.7, SD 2.1 [control group]; *P*=.008) and A-State (mean 36.7, SD 5.5 vs mean 42.3, SD 7.2; *P*<.001) scores after ablation. The BFI score after ablation was significantly lower in the intervention group than in the control group (mean 3.4, SD 2.3 vs mean 4.7, SD 2.2; *P*=.01).

**Table 2:** NRS^a^, A-State^b^, and BFI^c^ scores of the intervention and control groups.

Variable	Baseline				Post intervention		
	Intervention group, mean (SD)	Control group, mean (SD)	*P* value	Intervention group, mean (SD)		Control group, mean (SD)	*P* value
NRS score	0.3 (0.5)	0.4 (0.5)	.66	4.6 (1.7)		5.7 (2.1)	.008
A-State score	30.7 (4.4)	31.8 (6.2)	.39	36.7 (5.5)		42.3 (7.2)	<.001
BFI score	1.4 (1.7)	1.2 (1.5)	.49	3.4 (2.3)		4.7 (2.2)	.01

^a^NRS: numerical rating scale.

^b^A-State: State Anxiety Inventory.

^c^BFI: Brief Fatigue Inventory.

### Secondary Outcomes

**Figure 3:**
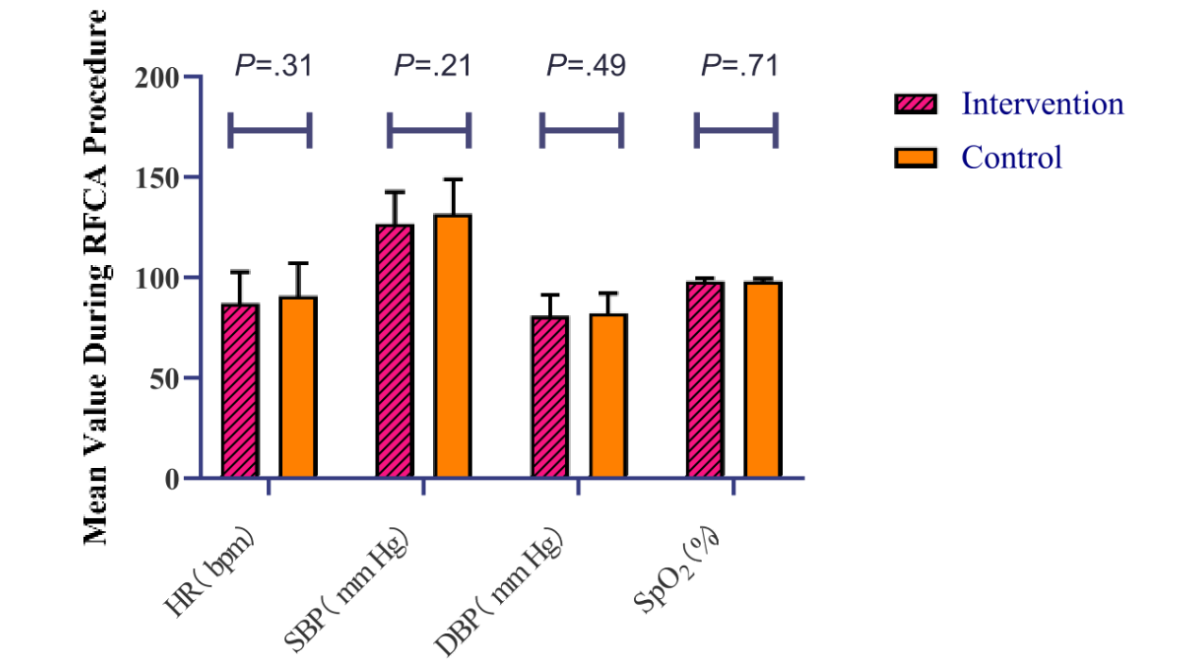
Hemodynamic parameters of the 2 groups. bpm: beats per minute; DBP: diastolic blood pressure; HR: heart rate; RFCA: radiofrequency catheter ablation; SBP: systolic blood pressure; SpO_2_: peripheral oxygen saturation.

Between the intervention and control groups in ablation, there were no significant differences in the mean heart rate (mean 87.4, SD 15.7 [intervention group] vs mean 91.1, SD 16.4 [control group] beats per minute; *P*=.31), systolic blood pressure (mean 127.2, SD 15.7 vs mean 131.9, SD 17.4 mm Hg; *P*=.21), diastolic blood pressure (mean 81.1, SD 10.5 vs mean 82.7, SD 9.6 mm Hg; *P*=.49), and SpO_2_ (mean 98.4%, SD 1.3% vs mean 98.5%, SD 1.2%; *P*=.71; Figure 3).

There were no significant differences in parecoxib and dexmedetomidine use between the intervention and control groups during ablation. The intervention group had significantly decreased fentanyl use compared to the control group (*P*=.003; Table 3). Additionally, patients in the intervention group reported significantly fewer times of pain than those in the control group during ablation (*P*<.001). The incidence of adverse events in the intervention group was lower than that in the control group, but the difference did not reach statistical significance (*P*=.15).

**Table 3:** Comparison of secondary outcomes between the intervention and control groups.

Medications and outcomes	Intervention group (n=40)	Control group (n=40)	*P* value
Parecoxib (mg), mean (SD)	29.0 (18.1)	27.0 (19.0)	.63
Fentanyl (mcg/kg), mean (SD)	3.96 (1.37)	4.85 (1.25)	.003
Dexmedetomidine (mcg/kg), mean (SD)	1.49 (0.82)	1.85 (1.00)	.57
Patient reports of pain (times), mean (SD)	1.5 (1.4)	2.8 (1.7)	<.001
Adverse events, n (%)	5 (13)	10 (25)	.15

## Discussion

### Principal Findings

The purpose of this study was to evaluate the effectiveness of a novel BCI-based mindfulness meditation app for patients with AF to improve their physical and psychological status in RFCA. The selected app provides EEG feedback for mindfulness meditation, which may help patients relax and reduce unpleasant experiences without interfering with the ablation process. The key findings showed significantly lower pain, anxiety, and fatigue scores in the intervention group than among those receiving conventional care. No significant differences were found in the mean heart rate, blood pressure, or SpO_2_ between groups during RFCA. Additionally, the intervention group had a significant decrease in fentanyl use, while the differences in other sedative drugs were not significant. Although the incidence of adverse events was lower in the intervention group, the difference was not significant.

Mobile health, via wireless technologies such as smartphone apps and wearable devices, is currently used for patients with AF mostly for screening, management, and rehabilitation [36-39]. Numerous studies have investigated the impact of meditation on cardiac disease and suggest that it may offer potential benefits for cardiovascular health, including reducing blood pressure, improving psychological and physiological responses to stress, and possibly mitigating AF progression by modulating the autonomic nervous system [21,40]. Mobile app–based mindfulness meditation has the potential to serve as an adjunct to the RFCA procedure for patients with AF owing to its low risks, potential benefits, and relatively low cost. Nevertheless, it is essential to acknowledge the costs associated with using a commercialized app and headband device, along with the requirement for labor and its cost to monitor the app. Additionally, caution should be exercised in making claims about the low risk of this intervention, as it is based solely on the findings of this small pilot study. Further research is needed to explore the relationship between meditation and AF ablation. To our knowledge, this is the first study to assess the effectiveness of a mindfulness meditation app together with a BCI-based wearable device for patients with AF during RFCA. This study achieved promising results and indicated that this type of intervention could be easily integrated into the standard RFCA workflow.

It is well known that RFCA for AF can be accompanied by considerable pain and anxiety when conscious sedation is used, which, however, has potential side effects [15]. Anxiety is common in patients with AF [41]. Patients may experience pain and uncertainty for several hours during RFCA, which could amplify negative emotions such as anxiety and made them feel exhausted. Therefore, it is necessary to monitor and intervene in patients with AF’s anxiety during ablation procedures [16]. Previous studies have indicated that apps based on nonpharmacological interventions could effectively reduce the fatigue, anxiety, pain, and the use of sedative drugs in invasive medical treatment [42-44]. Nørgaard et al [45] examined the effects of visualization together with usual pain medication in comparison with conventional care among patients with AF undergoing RFCA, and found significant reductions in the perception of pain and anxiety, as well as the doses of analgesics used in the intervention group [46]. Wearable devices are drastically changing medical practices nowadays. Roxburgh et al [47] implemented a virtual reality headset for patients with AF undergoing cryoballoon ablation under conscious sedation and found that the virtual reality group had a significantly lower perceived pain score and higher comfort score. In recent years, there has been a growing body of literature examining the effects of mindfulness training supported by EEG feedback. Crivelli et al [48] investigated the potential benefits of EEG-based brain-sensing device–supported mindfulness practices in individuals with mild stress levels and found a significant reduction in stress and anxiety. Similarly, Balconi et al [49] used mindfulness exercises in combination with wearable EEG information sensor devices to explore the effect of reducing overall stress levels in healthy individuals and found significant improvements in physiological (heart rate and variability) and subjective markers of stress (perceived stress, anxiety, and mood states). These findings are in line with those of this study, suggesting that EEG feedback may facilitate meditation by providing real-time information to aid users in achieving a mindfulness state. In our study, we assessed the effectiveness of an intervention based on a mindfulness meditation app combined with a BCI-based wearable device, which provides meditation guidance with synchronous EEG feedback to patients. This intervention has a minimal learning curve without requiring specialist training and provides personalized feedback, which may enhance patient engagement and adherence [50]. The potential mechanisms underlying pain, anxiety, and fatigue relief through mindfulness meditation are likely linked with the ability of meditation to change the activity of the insula, somatosensory cortex, anterior cingulate cortex, and prefrontal cortex. These changes may reduce patients' attention, memory, and perception of physical and psychological discomfort [51,52].

When patients experience physical and mental discomfort in RFCA, the levels of catecholamines, adrenocorticotropic hormone, prolactin, cortisol, and prostaglandins in their blood may increase [53], which could result in unstable hemodynamic parameters (heart rate, blood pressure, and SpO_2_) [54] and, thereby, affect the performance of procedures and patient safety. Previous studies have indicated that nonpharmacological interventions could effectively stabilize hemodynamic parameters in invasive operative procedures [42,55]. In this study, however, the between-group differences in mean heart rate, blood pressure, or SpO_2_ during RFCA did not reach statistical significance. The observed discrepancy between objective and subjective outcomes may be attributed to various factors. One possible explanation is that blood pressure changes induced by meditation through the autonomic nervous system may be a long-term process [21]. Furthermore, the timing of data collection may have influenced the findings, as objective measures were collected during the intervention, whereas subjective data were gathered through self-reports post intervention. It is worth noting that patients with AF frequently experience AF episodes during RFCA procedures, which may lead to variations in heart rate and blood pressure levels and contribute to the lack of significant differences between the groups. Additional research with larger sample sizes may help elucidate the potential impact of the intervention on these objective parameters. Although there was no significant difference, the incidence of adverse events was lower in the intervention group. This indicates a possibility for the potential protective effects of our intervention during RFCA, which merit further investigations.

### Strengths and Limitations

A strength of this study is that it is the first randomized controlled trial, to our knowledge, to explore the effectiveness of a mindfulness meditation app together with a BCI-based wearable device among patients with AF during RFCA, which adds to the evidence base in the areas of meditation and mobile health. This study was designed rigorously. We provided the same care protocol and implemented the use of wearable devices for both groups, and recorded the time, energy, and temperature of ablation to ensure comparable conditions. In addition, this study was performed in a pragmatic setting and no maximum age for participation was stated, which adds to the generalizability of our findings.

This study also has some limitations. First, it was not a double-blind trial. Neither study staff nor patients were blinded to the intervention due to the nature of the study design. However, the data analysts were masked to group allocation. Additionally, meditation practice is a long-term process. Even with the help of apps and wearable devices, it takes time to master meditation techniques to reach a meditative state faster. At least one preoperative practice session could be added to the protocol for the intervention group; however, this might affect the comparability of the baseline measures. Lastly, this is a single-center study, thus limiting the generalizability of our findings.

### Conclusions

In conclusion, this study shows that BCI-based app–delivered mindfulness meditation significantly relieved pain, anxiety, fatigue, and may reduce the doses of sedative medication used during RFCA for AF. Although no significant differences in hemodynamic parameters and the incidence of adverse events were observed, there was a decrease in the incidence of adverse events in the intervention group. Smartphone apps and wearable devices could serve as feasible and promising adjuncts to improve patients with AF’s experience in RFCA.

## Appendix

Multimedia Appendix 1 CONSORT-eHEALTH checklist (V 1.6.1).

## Abbreviations

AF: atrial fibrillation
A-State: State Anxiety Inventory
BCI: brain-computer interface
BFI: Brief Fatigue Inventory
CONSORT: Consolidated Standards of Reporting Trials
EEG: electroencephalography
RFCA: radiofrequency catheter ablation
SpO^2^: peripheral oxygen saturation

## References

[1] Hindricks G, Potpara T, Dagres N, Arbelo E, Bax JJ, Blomström-Lundqvist C, Boriani G, Castella M, Dan G, Dilaveris PE, Fauchier L, Filippatos G, Kalman JM, Meir ML, Lane DA, Lebeau J, Lettino M, Lip GY, Pinto FJ, Neil Thomas G, Valgimigli M, Van Gelder IC, Van Putte BP, Watkins CL (2021). 2020 ESC Guidelines for the diagnosis and management of atrial fibrillation developed in collaboration with the European Association for Cardio-Thoracic Surgery (EACTS). Revista Española de Cardiología (English Edition).

[2] January Craig T, Wann L Samuel, Calkins Hugh, Chen Lin Y, Cigarroa Joaquin E, Cleveland Joseph C, Ellinor Patrick T, Ezekowitz Michael D, Field Michael E, Furie Karen L, Heidenreich Paul A, Murray Katherine T, Shea Julie B, Tracy Cynthia M, Yancy Clyde W (2019). 2019 AHA/ACC/HRS focused update of the 2014 AHA/ACC/HRS guideline for the management of patients with atrial fibrillation: a report of the American College of Cardiology/American Heart Association Task Force on clinical practice guidelines and the Heart Rhythm Society. J Am Coll Cardiol.

[3] Magnussen C, Niiranen Teemu J, Ojeda Francisco M, Gianfagna Francesco, Blankenberg Stefan, Njølstad Inger, Vartiainen Erkki, Sans Susana, Pasterkamp Gerard, Hughes Maria, Costanzo Simona, Donati Maria Benedetta, Jousilahti Pekka, Linneberg Allan, Palosaari Tarja, de Gaetano Giovanni, Bobak Martin, den Ruijter Hester M, Mathiesen Ellisiv, Jørgensen Torben, Söderberg Stefan, Kuulasmaa Kari, Zeller Tanja, Iacoviello Licia, Salomaa Veikko, Schnabel Renate B, BiomarCaRE Consortium (2017). Sex differences and aimilarities in atrial fibrillation epidemiology, risk factors, and mortality in community cohorts: results from the BiomarCaRE Consortium (Biomarker for Cardiovascular Risk Assessment in Europe). Circulation.

[4] Wang L, Ze F, Li J, Mi L, Han B, Niu H, Zhao N (2021). Trends of global burden of atrial fibrillation/flutter from Global Burden of Disease Study 2017. Heart.

[5] Sheikh A, Patel NJ, Nalluri N, Agnihotri K, Spagnola J, Patel A, Asti D, Kanotra R, Khan H, Savani C, Arora S, Patel N, Thakkar B, Patel N, Pau D, Badheka AO, Deshmukh A, Kowalski M, Viles-Gonzalez J, Paydak H (2015). Trends in hospitalization for atrial fibrillation: epidemiology, cost, and implications for the future. Prog Cardiovasc Dis.

[6] Rahman F, Kwan GF, Benjamin EJ (2014). Global epidemiology of atrial fibrillation. Nat Rev Cardiol.

[7] Chew DS, Li Y, Cowper PA, Anstrom KJ, Piccini JP, Poole JE, Daniels MR, Monahan KH, Davidson-Ray L, Bahnson TD, Al-Khalidi HR, Lee KL, Packer DL, Mark DB (2022). Cost-effectiveness of catheter ablation versus antiarrhythmic drug therapy in atrial fibrillation: the CABANA randomized clinical trial. Circulation.

[8] Liu Y, Liu Q, Yang Y, Zhang C, Yin H, Wu J, Yao L, Jin L, Yang J, Feng L, Xie R (2022). Effect of radiofrequency catheter ablation on left atrial structure and function in patients with different types of atrial fibrillation. Sci Rep.

[9] Morillo CA, Verma A, Connolly SJ, Kuck KH, Nair GM, Champagne J, Sterns LD, Beresh H, Healey JS, Natale A, RAAFT-2 Investigators (2014). Radiofrequency ablation vs antiarrhythmic drugs as first-line treatment of paroxysmal atrial fibrillation (RAAFT-2): a randomized trial. JAMA.

[10] Shi S, Tang Y, Zhao Q, Yan H, Yu B, Zheng Q, Li Y, Zheng L, Yuan Y, Zhong J, Xu J, Wu Y, Xu J, Chen L, Li S, Jiang J, Wang J, Fan J, Chen M, Tang B, Li W, Wu Q, Shi B, Zhou S, Zhao X, Yin Y, Zhang Z, Zhong G, Han X, Liu F, Wu M, Gao L, Yang B, Huang H, Huang C, China Atrial Fibrillation Center Project Team) (2022). Prevalence and risk of atrial fibrillation in China: a national cross-sectional epidemiological study. Lancet Reg Health West Pac.

[11] Writing committee of the report on cardiovascular health and diseases in China (2022). Report on cardiovascular health and diseases in China 2021: an updated summary. Biomed Environ Sci.

[12] Wang Z, Jia L, Shi T, Liu C (2021). General anesthesia is not superior to sedation in clinical outcome and cost-effectiveness for ablation of persistent atrial fibrillation. Clin Cardiol.

[13] Hummel J, Elsayed-Awad H (2011). Walking the tightrope between deep sedation and general anesthesia: by whom can this safely be done?. J Cardiovasc Electrophysiol.

[14] Di Biase L, Saenz LC, Burkhardt DJ, Vacca M, Elayi CS, Barrett CD, Horton R, Bai R, Siu A, Fahmy TS, Patel D, Armaganijan L, Wu CT, Kai S, Ching CK, Phillips K, Schweikert RA, Cummings JE, Arruda M, Saliba WI, Dodig M, Natale A (2009). Esophageal capsule endoscopy after radiofrequency catheter ablation for atrial fibrillation. Circ: Arrhythmia and Electrophysiology.

[15] Smith HS, Laufer A (2014). Opioid induced nausea and vomiting. Eur J Pharmacol.

[16] Koleck TA, Mitha SA, Biviano A, Caceres BA, Corwin EJ, Goldenthal I, Creber RM, Turchioe MR, Hickey KT, Bakken S (2021). Exploring depressive symptoms and anxiety among patients with atrial fibrillation and/or flutter at the time of cardioversion or ablation. J Cardiovasc Nurs.

[17] Wasserlauf J, Kaplan RM, Walega DR, Arora R, Chicos AB, Kim SS, Lin AC, Verma N, Patil KD, Knight BP, Passman RS (2020). Patient-reported outcomes after cryoballoon ablation are equivalent between moderate sedation and general anesthesia. J Cardiovasc Electrophysiol.

[18] Attanasio Philipp, Huemer Martin, Shokor Parwani Abdul, Boldt Leif-Hendrik, Mügge Andreas, Haverkamp Wilhelm, Wutzler Alexander (2016). Pain reactions during pulmonary vein isolation under deep sedation: cryothermal versus radiofrequency ablation. Pacing Clin Electrophysiol.

[19] Nørgaard Marianne W, Pedersen PU, Bjerrum M (2015). Visualisation during ablation of atrial fibrillation - stimulating the patient's own resources: patients' experiences in relation to pain and anxiety during an intervention of visualisation. Eur J Cardiovasc Nurs.

[20] Lang EV, Benotsch EG, Fick LJ, Lutgendorf S, Berbaum ML, Berbaum KS, Logan H, Spiegel D (2000). Adjunctive non-pharmacological analgesia for invasive medical procedures: a randomised trial. Lancet.

[21] Levine GN, Lange RA, Bairey‐Merz CN, Davidson RJ, Jamerson K, Mehta PK, Michos ED, Norris K, Ray IB, Saban KL, Shah T, Stein R, Smith SC (2017). Meditation and cardiovascular risk reduction. JAHA.

[22] Izgu N, Gok Metin Z, Karadas C, Ozdemir L, Metinarikan N, Corapcıoglu D (2020). Progressive muscle relaxation and mindfulness meditation on neuropathic pain, fatigue, and quality of life in patients with type 2 diabetes: a randomized clinical trial. J Nurs Scholarsh.

[23] Thomas Z, Novak M, Platas SGT, Gautier M, Holgin AP, Fox R, Segal M, Looper KJ, Lipman M, Selchen S, Mucsi I, Herrmann N, Rej S (2017). Brief mindfulness meditation for depression and anxiety symptoms in patients undergoing hemodialysis. CJASN.

[24] Mani M, Kavanagh DJ, Hides L, Stoyanov SR (2015). Review and evaluation of mindfulness-based iPhone apps. JMIR Mhealth Uhealth.

[25] Mridha MF, Das SC, Kabir MM, Lima AA, Islam MR, Watanobe Y (2021). Brain-computer interface: advancement and challenges. Sensors (Basel).

[26] Daly JJ, Huggins JE (2015). Brain-computer interface: current and emerging rehabilitation applications. Arch Phys Med Rehabil.

[27] Torres EP, Torres EA, Hernández-Álvarez Myriam, Yoo SG (2020). EEG-based BCI emotion recognition: a survey. Sensors (Basel).

[28] Devipriya A, Nagarajan N (2018). A novel method of segmentation and classification for meditation in health care systems. J Med Syst.

[29] Pronk Y, Peters MCWM, Sheombar A, Brinkman J (2020). Effectiveness of a mobile eHealth app in guiding patients in pain control and opiate use after total knee replacement: randomized controlled trial. JMIR Mhealth Uhealth.

[30] Jibb LA, Stevens BJ, Nathan PC, Seto E, Cafazzo JA, Johnston DL, Hum V, Stinson JN (2017). Implementation and preliminary effectiveness of a real-time pain management smartphone app for adolescents with cancer: A multicenter pilot clinical study. Pediatr Blood Cancer.

[31] Smith JL, Allen JW, Haack C, Wehrmeyer K, Alden K, Lund MB, Mascaro JS (2021). The impact of app-delivered mindfulness meditation on functional connectivity and self-reported mindfulness among health profession trainees. Mindfulness (N Y).

[32] Chiu LYL, Sun T, Ree R, Dunsmuir D, Dotto A, Ansermino JM, Yarnold C (2019). The evaluation of smartphone versions of the visual analogue scale and numeric rating scale as postoperative pain assessment tools: a prospective randomized trial. Can J Anaesth.

[33] Blödt Susanne, Pach D, Eisenhart-Rothe SV, Lotz F, Roll S, Icke K, Witt CM (2018). Effectiveness of app-based self-acupressure for women with menstrual pain compared to usual care: a randomized pragmatic trial. Am J Obstet Gynecol.

[34] Liu W, Mao Y, Wei D, Yang J, Du X, Xie P, Qiu J (2016). Structural asymmetry of dorsolateral prefrontal cortex correlates with depressive symptoms: evidence from healthy individuals and patients with major depressive disorder. Neurosci Bull.

[35] Park S, Sato Y, Takita Y, Tamura N, Ninomiya A, Kosugi T, Sado M, Nakagawa A, Takahashi M, Hayashida T, Fujisawa D (2020). Mindfulness-based cognitive therapy for psychological distress, fear of cancer recurrence, fatigue, spiritual well-being, and quality of life in patients with breast cancer-a randomized controlled trial. J Pain Symptom Manage.

[36] Rizas KD, Freyer L, Sappler N, von Stülpnagel Lukas, Spielbichler P, Krasniqi A, Schreinlechner M, Wenner FN, Theurl F, Behroz A, Eiffener E, Klemm MP, Schneidewind A, Zens M, Dolejsi T, Mansmann U, Massberg S, Bauer A (2022). Smartphone-based screening for atrial fibrillation: a pragmatic randomized clinical trial. Nat Med.

[37] Cai C, Bao Z, Wu N, Wu F, Sun G, Yang G, Chen M (2022). A novel model of home-based, patient-tailored and mobile application-guided cardiac telerehabilitation in patients with atrial fibrillation: A randomised controlled trial. Clin Rehabil.

[38] Guo Y, Guo J, Shi X, Yao Y, Sun Y, Xia Y, Yu B, Liu T, Chen Y, Lip GYH, mAF-App II Trial investigators (2020). Mobile health technology-supported atrial fibrillation screening and integrated care: a report from the mAFA-II trial Long-term Extension Cohort. Eur J Intern Med.

[39] Perez MV, Mahaffey KW, Hedlin H, Rumsfeld JS, Garcia A, Ferris T, Balasubramanian V, Russo AM, Rajmane A, Cheung L, Hung G, Lee J, Kowey P, Talati N, Nag D, Gummidipundi SE, Beatty A, Hills MT, Desai S, Granger CB, Desai M, Turakhia MP (2019). Large-scale assessment of a smartwatch to identify atrial fibrillation. N Engl J Med.

[40] Bashir M Usmaan, Bhagra Anjali, Kapa Suraj, McLeod Christopher J (2019). Modulation of the autonomic nervous system through mind and body practices as a treatment for atrial fibrillation. Rev Cardiovasc Med.

[41] Thrall G, Lip GY, Carroll D, Lane D (2007). Depression, anxiety, and quality of life in patients with atrial fibrillation. Chest.

[42] Guerrier G, Abdoul H, Jilet L, Rothschild P, Baillard C (2021). Efficacy of a web app-based music intervention during cataract surgery: a randomized clinical trial. JAMA Ophthalmol.

[43] Huberty J, Puzia ME, Green J, Vlisides-Henry RD, Larkey L, Irwin MR, Vranceanu A (2021). A mindfulness meditation mobile app improves depression and anxiety in adults with sleep disturbance: Analysis from a randomized controlled trial. Gen Hosp Psychiatry.

[44] Chai PR, Schwartz E, Hasdianda MA, Azizoddin DR, Kikut A, Jambaulikar GD, Edwards RR, Boyer EW, Schreiber KL (2020). A brief music app to address pain in the emergency department: prospective study. J Med Internet Res.

[45] Nørgaard Marianne W, Werner Anette, Abrahamsen Randi, Larsen Birgitte, Darmer Mette Rosendal, Pedersen Preben U (2013). Visualization and attentive behavior for pain reduction during radiofrequency ablation of atrial fibrillation. Pacing Clin Electrophysiol.

[46] Nørgaard Marianne W, Pedersen PU, Bjerrum M (2018). Understanding how patients use visualization during ablation of atrial fibrillation in reducing their experience of pain, anxiety, consumption of pain medication and procedure length: Integrating quantitative and qualitative results. Appl Nurs Res.

[47] Roxburgh T, Li A, Guenancia C, Pernollet P, Bouleti C, Alos B, Gras M, Kerforne T, Frasca D, Le Gal F, Christiaens L, Degand B, Garcia R (2021). Virtual reality for sedation during atrial fibrillation ablation in clinical practice: observational study. J Med Internet Res.

[48] Crivelli D, Fronda G, Venturella I, Balconi M (2018). Supporting mindfulness practices with brain-sensing devices. Cognitive and electrophysiological evidences. Mindfulness.

[49] Balconi M, Fronda G, Crivelli D (2019). Effects of technology-mediated mindfulness practice on stress: psychophysiological and self-report measures. Stress.

[50] Barello S, Triberti S, Graffigna G, Libreri C, Serino S, Hibbard J, Riva G (2015). eHealth for patient engagement: a systematic review. Front Psychol.

[51] Zeidan F, Vago D (2016). Mindfulness meditation-based pain relief: a mechanistic account. Ann N Y Acad Sci.

[52] Tang Y, Hölzel Britta K, Posner MI (2015). The neuroscience of mindfulness meditation. Nat Rev Neurosci.

[53] Foji S, Tadayonfar MA, Mohsenpour M, Rakhshani MH (2015). The study of the effect of guided imagery on pain, anxiety and some other hemodynamic factors in patients undergoing coronary angiography. Complement Ther Clin Pract.

[54] Bayrak A, Sagiroglu G, Copuroglu E (2019). Effects of preoperative anxiety on intraoperative hemodynamics and postoperative pain. J Coll Physicians Surg Pak.

[55] Angioli R, De Cicco Nardone C, Plotti F, Cafà Ester Valentina, Dugo N, Damiani P, Ricciardi R, Linciano F, Terranova C (2014). Use of music to reduce anxiety during office hysteroscopy: prospective randomized trial. J Minim Invasive Gynecol.

